# Prediction of Residual Disease in Unresectable Head and Neck Squamous Cell Carcinoma After Concurrent Chemoradiotherapy

**DOI:** 10.14740/wjon2821

**Published:** 2026-07-30

**Authors:** Chawalit Lakdee, Jayanton Patumanond, Peeraphong Thiarawat, Thanin Lokeskrawee, Suppachai Lawanaskol, Piradee Chinati, Wanwisa Bumrungpagdee, Suwapim Chanlaor

**Affiliations:** a Department of Radiology, Buddhachinaraj Phitsanulok Hospital, Phitsanulok 65000, Thailand; b Clinical Epidemiology and Clinical Statistics Unit, Faculty of Medicine, Naresuan University, Phitsanulok 65000, Thailand; c Department of Surgery, Faculty of Medicine, Naresuan University, Phitsanulok 65000, Thailand; d Department of Emergency Medicine, Lampang Hospital, Lampang 52000, Thailand; e Chaiprakarn Hospital, Chiang Mai 50320, Thailand; f Department of Otolaryngology–Head and Neck Surgery, Buddhachinaraj Phitsanulok Hospital, Phitsanulok 65000, Thailand

**Keywords:** Squamous cell carcinoma of head and neck, Residual disease, Time-to-treatment, Neutrophil-to-lymphocyte ratio, Inflammatory biomarkers, Body mass index

## Abstract

**Background:**

Concurrent chemoradiotherapy (CCRT) for unresectable head and neck cancer (HNC) in advanced stages is associated with a high probability of residual disease (RD). Salvage surgery after completing CCRT often leads to severe complications, and palliative care is required if surgery is not feasible. The aim of this study was to develop a prediction model for RD based on pretreatment clinical features.

**Methods:**

This retrospective observational cohort study analyzed the medical records of patients with unresectable HNC who received CCRT at the Radiation Oncology Unit of Buddhachinaraj Phitsanulok Hospital, Thailand, between October 2019 and October 2025. Clinical features and laboratory results were collected before treatment. Tumor response was evaluated 8–12 weeks after completion of CCRT using computed tomography or magnetic resonance imaging scans according to the Response Evaluation Criteria in Solid Tumors. Multivariable logistic regression was used to identify predictors of RD. The model performance was evaluated for discrimination using the area under the receiver operating characteristic curve (AuROC) and for calibration using calibration plots. Internal validation was performed using the bootstrap resampling method. Decision curve analysis was also performed.

**Results:**

A total of 382 patients with HNC were included. After the completion of CCRT, 180 patients (47.1%) presented with RD. The final model retained primary tumor site (hypopharynx (odds ratio (OR): 3.56; 95% confidence interval (CI): 1.10–11.40) and oral cavity (OR: 3.77; 95% CI: 0.95–14.90)), maximum tumor diameter (OR: 3.37; 95% CI: 2.59–4.39), time to initiation of radiotherapy (OR: 1.01; 95% CI: 1.00–1.02), body mass index (OR: 0.97; 95% CI: 0.88–1.06), neutrophil-to-lymphocyte ratio (OR: 1.02; 95% CI: 0.94–1.10), and hemoglobin level (OR: 0.94; 95% CI: 0.79–1.11). The model demonstrated excellent discrimination, with an AuROC of 0.929 (95% CI: 0.903–0.954). At an RD probability cutoff of ≥ 47.1%, the model yielded a positive predictive value of 81.2% (95% CI: 75.1–86.4) and a negative predictive value of 89.2% (95% CI: 83.8–93.3).

**Conclusions:**

This clinical feature-based prediction model accurately predicted the probability of RD in patients with unresectable HNC. Physicians can utilize this model for pre-CCRT treatment planning to identify high-risk patients.

## Introduction

In patients with locoregionally advanced head and neck squamous cell carcinoma (HNSCC), concurrent chemoradiotherapy (CCRT) significantly improves overall survival (OS) compared with radiotherapy alone, whereas induction or adjuvant chemotherapy provides no significant survival benefit [1]. Although CCRT is the standard treatment for advanced HNSCC, approximately 50% of patients experience disease relapses after treatment [2]. The presence of residual disease (RD) is clinically significant, as patients receiving palliative treatment have a 5-year OS of 0% compared with 28% after salvage surgery [2]. Salvage surgery, such as salvage neck dissection, is associated with significant complications, including grade 3 toxicity. Accordingly, risk stratification of patients prior to CCRT is critical for reducing RD and the need for high-risk surgical interventions [3]. However, most prognostic factors studied prior to CCRT appear to predict OS and disease-free survival, as opposed to RD. High levels of pretreatment inflammatory markers, such as the platelet-to-lymphocyte ratio (PLR) and neutrophil-to-lymphocyte ratio (NLR), are adversely associated with OS [4]. Human papillomavirus (HPV)-positive oropharyngeal cancer has better OS, even after disease progression [5]. To our knowledge, few studies have investigated the association between pretreatment prognostic factors in patients with head and neck cancer (HNC) receiving CCRT and early treatment responses, including RD and locoregional control. For example, although tumor repopulation, such as a growth rate of 3.2% per day and a volume doubling time of 19 days, has been quantified, comprehensive pretreatment evaluation remains limited [6]. Good clinical performance status and the use of intensity-modulated radiation therapy (IMRT) have been associated with improved local control [7]. Moreover, risk stratification can be enhanced by advanced imaging, and MRI-based tumor volume assessment has been shown to predict local failure [8], and modern deep learning algorithms based on MRI data have recently demonstrated their ability to assess RD, thus supporting the selection of treatment [9]. Therefore, the aim of this study was to develop and validate a clinical prediction model based on pretreatment clinical characteristics to predict treatment response in patients with unresectable, advanced HNSCC prior to the initiation of CCRT.

## Materials and Methods

### Study design and setting

A retrospective study was conducted to establish a clinical risk prediction model for RD after CCRT in patients with unresectable HNC. Patients treated with IMRT using helical tomotherapy at the radiation therapy unit of Buddhachinaraj Phitsanulok Hospital between October 2019 and October 2025 were evaluated in the present study. Patients were ≥ 18 years of age with unresectable HNC and underwent CCRT with cisplatin-based chemotherapy. All enrolled patients had histologically confirmed squamous cell carcinoma (SCC) and unresectable disease. Clinical staging was performed using magnetic resonance imaging (MRI) or computed tomography (CT). The exclusion criteria were as follows: (1) previous radiotherapy, (2) chemotherapy prior to CCRT, and (3) distant metastasis at initial staging. Supportive procedures such as tracheostomy and prophylactic feeding-tube insertion were not exclusion criteria; patients who underwent these procedures were included, as they are part of routine supportive management in unresectable HNC. Any delay related to such procedures was captured within the time to initiation of radiotherapy, which was measured from the date of biopsy to the first day of CCRT.

### Treatment

The patients received IMRT with helical tomotherapy. A total radiation dose of 70–72 Gy was administered to the gross tumor volume (GTV), whereas clinical target volumes (CTVs) covering regions of subclinical disease received doses of 44–63 Gy. Radiotherapy was given using a standard fractionation schedule of five fractions per week. All patients received cisplatin (100 mg/m^2^) intravenous chemotherapy on days 1, 22, and 43 of radiotherapy.

### Outcome and definition of variables

The clinical endpoint was evaluated using MRI or CT scanning 8–12 weeks after CCRT. Response assessment was conducted following the Response Evaluation Criteria in Solid Tumors (RECIST), version 1.1 [10]. The main outcome, termed RD, was characterized by any response other than a complete response (CR), such as a partial response (PR), stable disease (SD), or progressive disease (PD). According to the RECIST 1.1 criteria, CR was defined as the disappearance of all tumors and a reduction in lymph nodes to < 10 mm on the short axis. PR was defined as a ≥ 30% decrease in the sum of the longest target lesion diameters. For PD, a ≥ 20% increase and an absolute increase of ≥ 5 mm compared with the smallest recorded sum were required to define PD. Lesions that did not qualify for PR or PD were classified as SD. To minimize bias, all candidate predictors judged in the final prediction model were sourced only from pretreatment records, and the extraction of baseline variables was conducted by researchers blinded to the ultimate clinical outcomes. RD was defined as a composite locoregional outcome at the 8- to 12-week posttreatment assessment, including any response other than CR (i.e., PR, SD, or PD) at the primary site and/or regional lymph nodes.

Pretreatment predictors and baseline clinical characteristics were collected from medical records. The variables were as follows: demographics (sex, age, and body mass index (BMI)), past medical history and comorbidities (diabetes mellitus, hypertension, and dyslipidemia), lifestyle factors (smoking and alcohol consumption), and tumor-specific characteristics (primary tumor site, histologic differentiation grade, tumor stage (1–4), nodal stage (1–3), and HPV status). Imaging-based data on tumor size at baseline were also recorded (e.g., anteroposterior, transverse, vertical, and maximum tumor sizes). Finally, baseline laboratory characteristics (hemoglobin, albumin, and NLR) were recorded. Time to initiation of radiotherapy was defined as the period from the date of tissue biopsy to the first date of start of CCRT.

### Specific time point for prediction

These assessments were performed during the initial consultation upon completion of clinical staging, using strict pretreatment data to predict the presence of RD. As part of the pretreatment assessment, all patients underwent baseline clinical and otolaryngologic examination, including endoscopic or flexible laryngoscopic evaluation where indicated.

### Study size estimation

Sample size estimation for the binary clinical prediction model was performed using pmsampsize [11]. Utilizing data from a pilot study at Buddhachinaraj Phitsanulok Hospital, which showed a prevalence of RD of 0.40 and an anticipated C-statistic of 0.85, it was determined that at least 369 participants were necessary to assess the six potential predictors of RD. This calculation guaranteed the inclusion of a minimum of 148 event cases. Although the cohort was defined by the study period, the available sample size was additionally assessed using pmsampsize. As the final cohort of 382 patients exceeded the estimated minimum requirement, the sample size was considered adequate for model development and to reduce the risk of overfitting.

### Statistical analysis

Statistical analyses were performed using Stata version 17.0. For categorical variables, descriptive statistics were reported as frequencies and percentages, and group comparisons were performed using Fisher’s exact tests. Continuous variables with normal distribution are reported as mean ± standard deviation (SD) and compared using the Student’s *t*-test. Conversely, continuous variables that were not normally distributed are presented as median (interquartile range (IQR)) and compared using the Wilcoxon rank-sum test. Missing data were minimal, with one missing value each for hemoglobin and the neutrophil-to-lymphocyte ratio. These missing values were replaced with the corresponding group mean values.

### Model development

Candidate predictors were initially assessed using univariable odds ratios (uORs). Multivariable logistic regression was used for model development to obtain the multivariable odds ratios (mORs) of the final retained variables. Manual predictor selection and multicollinearity assessment were performed to optimize predictive performance for RD. The area under the receiver operating characteristic curve (AuROC) and 95% confidence intervals (CIs) were computed to measure model discrimination. Calibration was thoroughly examined using a calibration plot, calibration slope, observed-to-expected (O:E) ratio, and calibration-in-the-large (CITL). Bootstrap resampling (200 repetitions) was used for internal validation. Decision curve analysis (DCA) was performed to estimate the net benefit (NB) across a range of threshold probabilities to assess clinical utility. For the categorical tumor-site variable, the supraglottic subsite was chosen as the reference category because it had an adequate sample size for stable estimation and provided a clinically interpretable baseline. This choice was made *a priori* for interpretability and does not affect model performance or predicted probabilities; it only changes the contrasts used for interpreting the other tumor-site categories.

### Establishment of the clinical cutoff point

The best clinical cutoff value for the predicted probability of RD was systematically determined from the final multivariable logistic regression model to optimize the predictive performance. In particular, the threshold at the linear predictor (linear combination of coefficients) was determined using a predicted probability equal to the overall prevalence of RD in this study cohort. Additionally, we performed a detailed evaluation of clinical utility by calculating the diagnostic performance metrics (sensitivity, specificity, positive predictive value (PPV), and negative predictive value (NPV)) across a wide range of predicted probability thresholds.

### Ethical approval

This study was registered with the Thai Clinical Trials Registry (TCTR; ID: TCTR20260322007). Ethics approval was obtained from the Institutional Review Board (IRB) of Buddhachinaraj Phitsanulok Hospital, Phitsanulok (Approval No. IRB/HREC No. 005/2569), and the Human Research Ethics Committee of Naresuan University (Approval No. P3-0017/2569). The requirement for informed consent was waived owing to the retrospective observational design. This study was conducted in compliance with the ethical standards of the responsible institutions on human subjects as well as with the Declaration of Helsinki.

## Results

**Figure 1:**
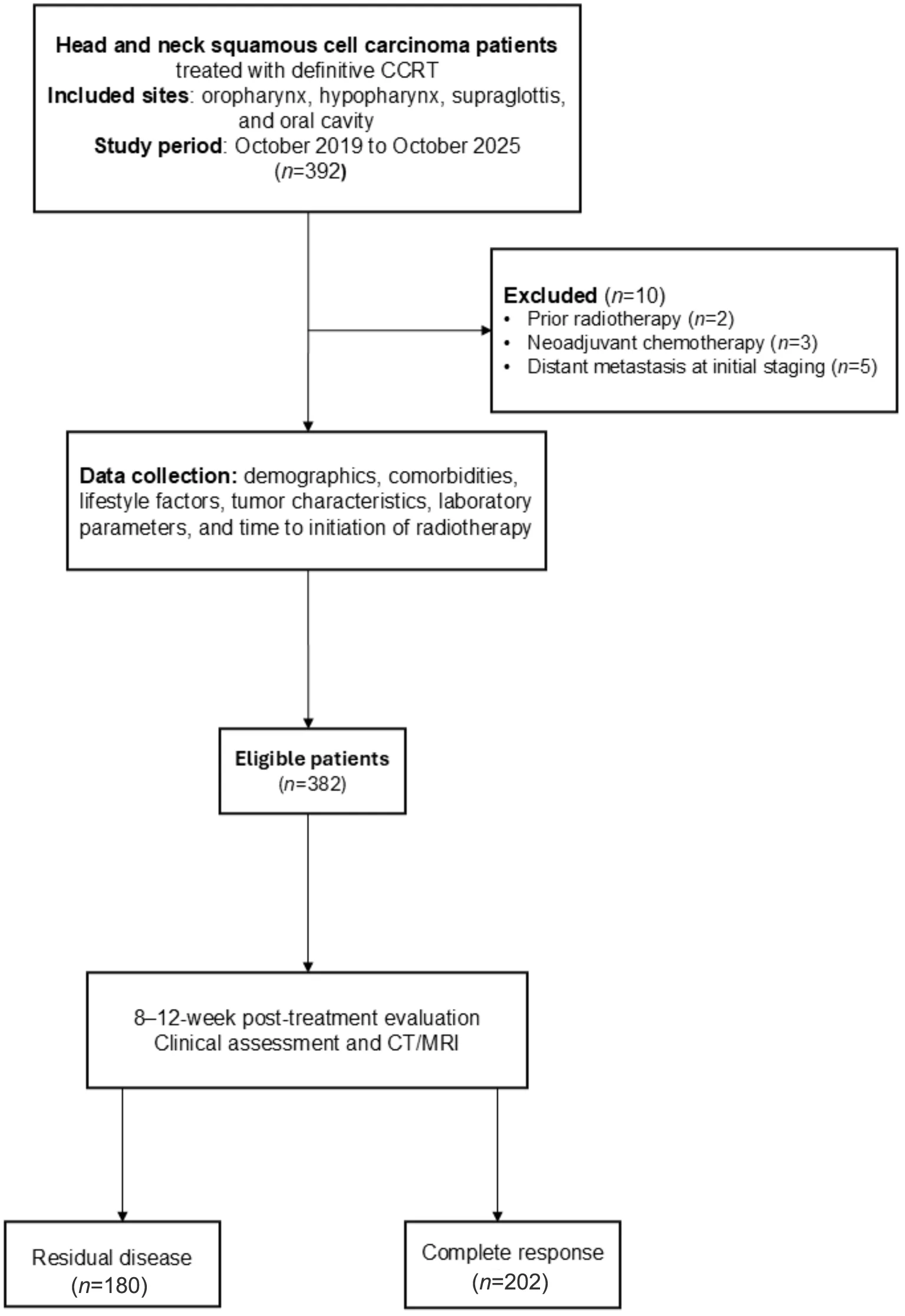
Study flow diagram. CCRT: concurrent chemoradiotherapy; CT: computed tomography; MRI: magnetic resonance imaging.

A total of 392 patients with HNC treated with definitive CCRT via image-guided helical tomotherapy were assessed. Ten patients were excluded because of prior radiotherapy (n = 2), neoadjuvant chemotherapy (n = 3), and distant metastasis at initial staging (n = 5). The final analysis cohort included 382 patients. At the 8- to 12-week clinical and radiographic assessment, 180 patients (47.1%) had RD, whereas 202 (52.9%) achieved a CR (Fig. 1). Among the 180 patients with RD, most had primary-site involvement, including 136 (75.6%) with RD confined to the primary site and 32 (17.8%) with both primary-site and regional nodal RD, whereas isolated regional nodal RD occurred in only 12 patients (6.7%).

### Baseline characteristics associated with treatment response (RD vs. CR)

In this study, the RD group was younger than the CR group, with mean ages of 55.9 ± 9.9 vs. 58.4 ± 11.0 years in the RD and CR groups, respectively (P = 0.018). Patients in the RD group had more advanced T-stage and nodal stage overall (both P < 0.001), with a higher proportion of T4 disease and N2–N3 nodal involvement. The maximum tumor diameter was significantly larger in the RD group (median 6.5 cm (IQR 5.7–7.3)) than in the CR group (median 3.5 cm (IQR 2.5–4.7), P < 0.001). Patients in the RD group had significantly lower mean hemoglobin (11.2 ± 1.9 vs. 12.2 ± 1.9 g/dL, P < 0.001), albumin (3.7 ± 0.5 vs. 3.9 ± 0.4 g/dL, P = 0.001), and BMI (18.9 ± 3.1 vs. 20.4 ± 3.9 kg/m^2^, P < 0.001) with a significantly higher median NLR compared with patients with CR (4.3 vs. 3.0; P < 0.001). Finally, HPV positivity was significantly less frequent in RD (7.6% vs. 41.4%, P = 0.001) (Table 1).

**Table 1:** Baseline Characteristics Associated With Treatment Response (Residual Disease vs. Complete Response)

Prognostic factors	Residual disease (n = 180), n (%)	Complete response (n = 202), n (%)	P value	AuROC
Male sex	75 (41.7)	91 (45.0)	0.536	0.516
Age (years), mean ± SD	55.9 ± 9.9	58.4 ± 11.0	0.018	0.585
Comorbidities				
Diabetes mellitus	8 (4.4)	11 (5.4)	0.814	0.505
Hypertension	23 (12.8)	36 (17.8)	0.202	0.525
Dyslipidemia	9 (5.0)	19 (9.4)	0.117	0.522
Smoking history	119 (66.1)	121 (59.9)	0.243	0.531
Alcohol consumption	111 (61.7)	122 (60.4)	0.834	0.506
Tumor site			< 0.001	0.683
Supraglottis	12 (6.7)	50 (24.8)		
Oropharynx	72 (40.0)	105 (52.0)		
Hypopharynx	70 (38.9)	35 (17.3)		
Oral cavity	26 (14.4)	12 (5.9)		
Histologic grade			0.698	0.522
Well differentiated	55 (30.8)	70 (34.7)		
Moderately differentiated	91 (50.8)	95 (47.0)		
Poorly differentiated	33 (18.4)	37 (18.3)		
Tumor stage (T-stage)			< 0.001	0.723
T1	8 (4.4)	17 (8.4)		
T2	18 (10.0)	53 (26.2)		
T3	35 (19.4)	86 (42.6)		
T4	119 (66.1)	46 (22.8)		
Nodal stage (N-stage)			< 0.001	0.689
N1	30 (16.7)	91 (45.0)		
N2	112 (62.2)	106 (52.5)		
N3	38 (21.1)	5 (2.5)		
Hemoglobin (g/dL), mean ± SD	11.2 ± 1.9	12.2 ± 1.9	< 0.001	0.641
Albumin (g/dL), mean ± SD	3.7 ± 0.5	3.9 ± 0.4	0.001	0.617
HPV-positive	6 (7.6)	53 (41.4)	0.001	0.669
NLR, median (IQR)	4.3 (2.7, 7.1)	3.0 (1.9, 4.4)	< 0.001	0.670
Maximum tumor diameter (cm), median (IQR)	6.5 (5.7, 7.3)	3.5 (2.5, 4.7)	< 0.001	0.914
Tumor size anteroposterior (cm), mean ± SD	4.4 ± 1.8	2.8 ± 1.3	< 0.001	0.761
Tumor size transverse (cm), mean ± SD	4.5 ± 1.7	2.7 ± 1.2	< 0.001	0.793
Tumor size vertical (cm), mean ± SD	5.7 ± 2.0	3.1 ± 1.5	< 0.001	0.850
Maximum lymph node diameter (cm), median (IQR)	2.0 (1.2, 5.0)	1.8 (1.0, 3.0)	0.169	0.563
BMI (kg/m^2^), mean ± SD	18.9 ± 3.1	20.4 ± 3.9	< 0.001	0.615
Time to initiation of radiotherapy (days), median (IQR)	62.0 (49.5, 84.5)	61.0 (52.0, 75.0)	0.217	0.538
Overall treatment time (days), mean ± SD	54.5 ± 10.8	52.5 ± 18.9	0.470	0.566

HPV testing was performed mainly in patients with oropharyngeal tumors. Histological grade was missing in one patient with residual disease. AuROC: area under the receiver operating characteristic curve; BMI: body mass index; HPV: human papillomavirus; IQR: interquartile range; NLR: neutrophil-to-lymphocyte ratio; SD: standard deviation.

### Model development and predictors of RD

The final multivariable model was derived using manual predictor selection, with multicollinearity assessed prior to model fitting. The retained predictors were tumor site, maximum tumor diameter, hemoglobin level, NLR, BMI, and time to initiation of radiotherapy. Among these, hypopharyngeal tumor site, larger maximum tumor diameter, and prolonged time to initiation of radiotherapy remained significant independent predictors of RD. Compared with supraglottic tumors, hypopharyngeal tumors were associated with higher odds of RD (OR: 3.56, 95% CI: 1.10–11.40; P = 0.033). In addition, a greater maximum tumor diameter was strongly associated with RD (OR: 3.37, 95% CI: 2.59–4.39; P < 0.001), whereas a longer interval to radiotherapy initiation was associated with a modest increase in the odds of RD (OR: 1.01, 95% CI: 1.00–1.02; P = 0.016) (Table 2).

**Table 2:** Model Development and Predictors of Residual Disease

Predictors	Univariable OR (95% CI)	P value	Multivariable OR (95% CI)	P value
Tumor site				
Supraglottis	1.00 (Reference)	-	1.00 (Reference)	-
Oropharynx	2.85 (1.42, 5.74)	0.003	1.39 (0.46, 4.20)	0.551
Hypopharynx	8.33 (3.93, 17.63)	< 0.001	3.56 (1.10, 11.40)	0.033
Oral cavity	9.02 (3.56, 22.88)	< 0.001	3.77 (0.95, 14.90)	0.058
Maximum tumor diameter (cm)	3.47 (2.72, 4.43)	< 0.001	3.37 (2.59, 4.39)	< 0.001
Hemoglobin (g/dL)	0.76 (0.68, 0.85)	< 0.001	0.94 (0.79, 1.11)	0.475
NLR	1.15 (1.07, 1.22)	< 0.001	1.02 (0.94, 1.10)	0.610
BMI (kg/m^2^)	0.88 (0.83, 0.94)	< 0.001	0.97 (0.88, 1.06)	0.519
Time to initiation of radiotherapy (days)	1.01 (1.00, 1.02)	0.005	1.01 (1.00, 1.02)	0.016

Overall multivariable model AuROC was 0.929. BMI: body mass index; CI: confidence interval; NLR: neutrophil-to-lymphocyte ratio; OR: odds ratio.

### Model performance and internal validation

**Figure 2:**
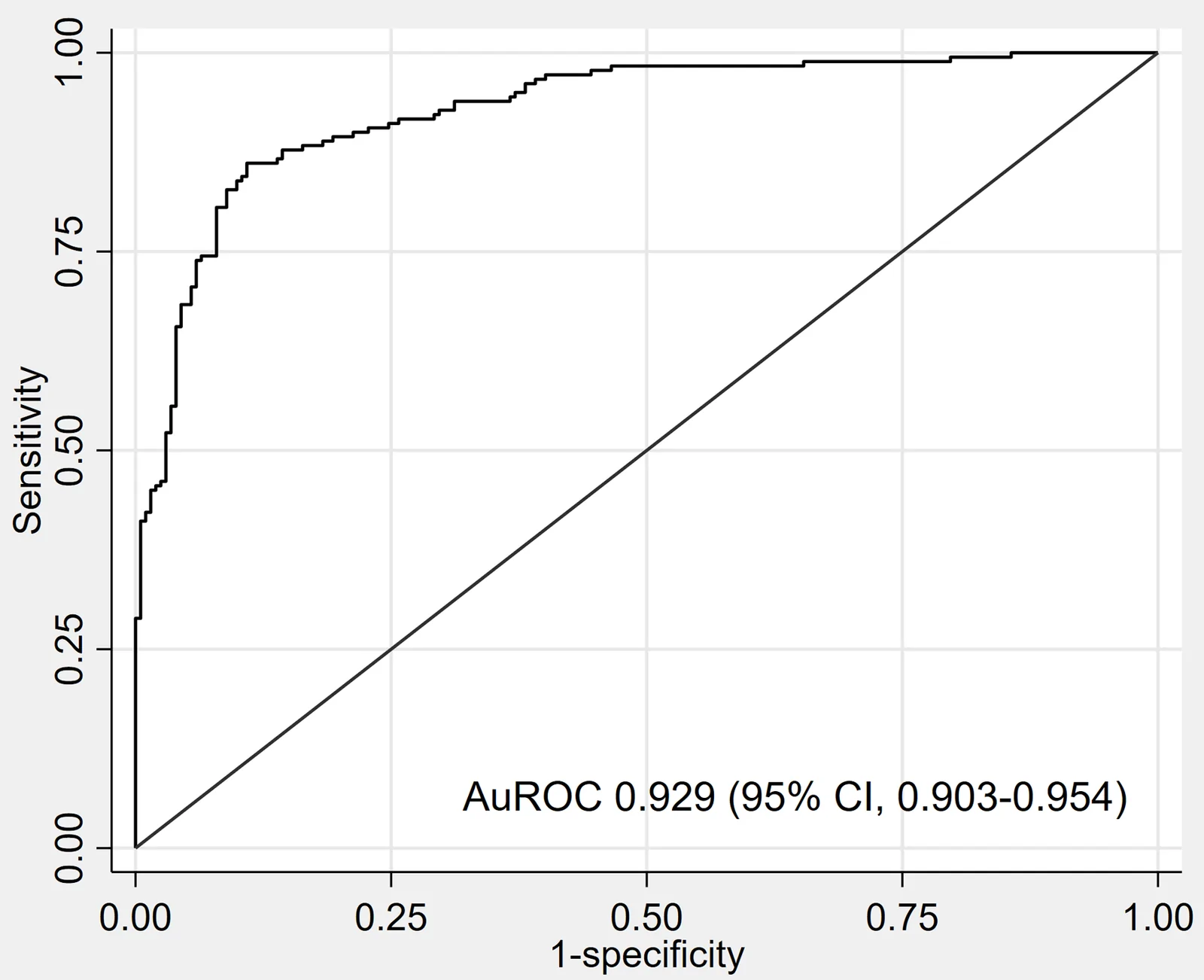
Area under the receiver operating characteristic curve (AuROC). CI: confidence interval.

**Figure 3:**
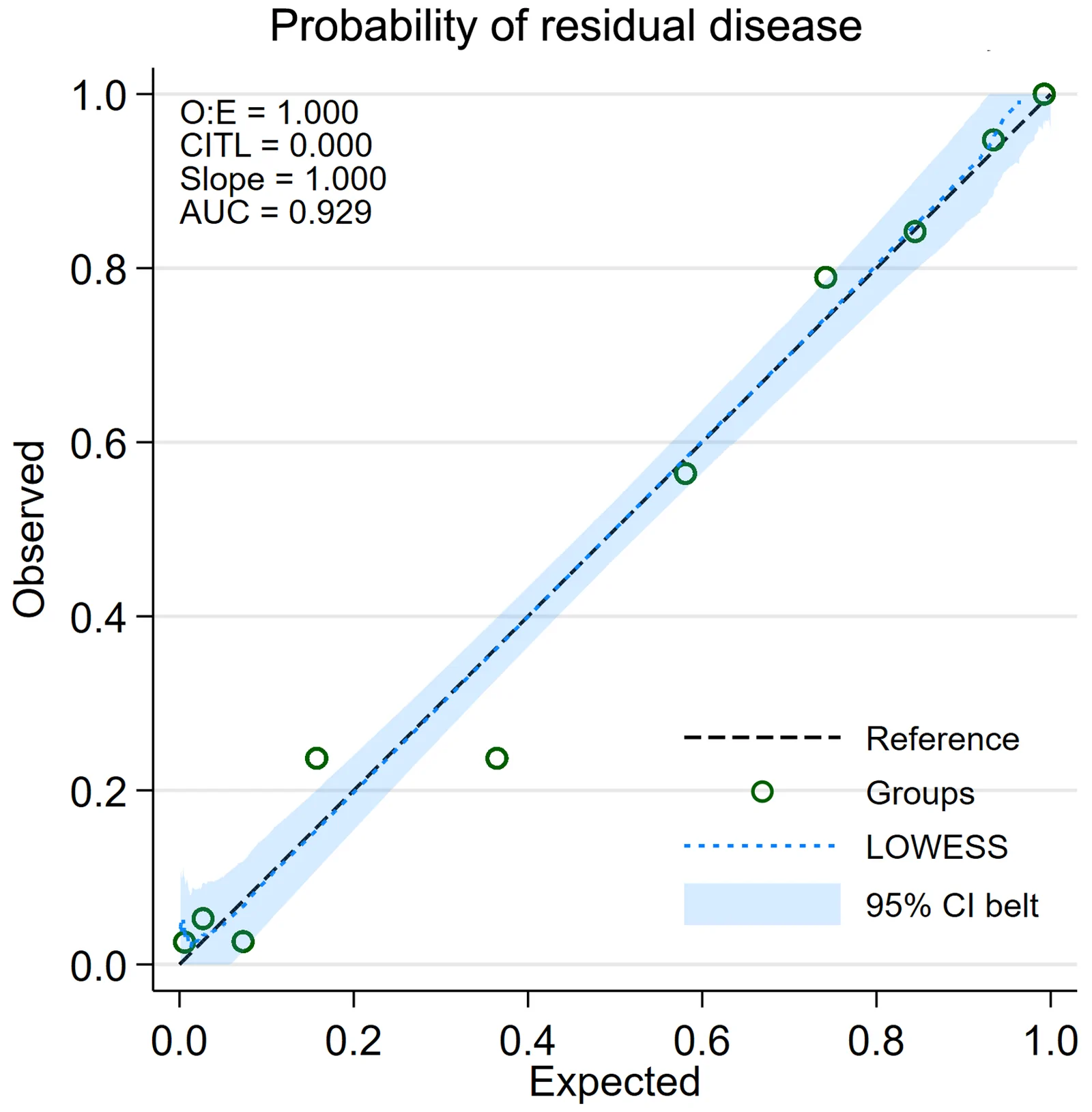
Calibration plot demonstrating the agreement between the predicted and observed probabilities of residual disease (RD). AUC shown in the plot is equivalent to AuROC. O:E ratio: observed-to-expected ratio; CITL: calibration-in-the-large.

The final clinical prediction model produced an excellent AuROC of 0.929. The apparent C-statistic was 0.929 (95% CI: 0.903–0.954) and showed a similar performance upon internal bootstrap validation (C-statistic, 0.929; 95% CI: 0.905–0.956). The model showed ideal calibration, with an observed-to-expected (O:E) ratio of 1.000 (95% CI: 0.930–1.070), a CITL of 0.000, and a calibration slope of 1.000 (Table 3). The final clinical prediction model showed excellent discrimination on the ROC curve (Fig. 2) and good calibration on the calibration plot (Fig. 3).

**Table 3:** Model Performance and Internal Validation

Parameters	Apparent performance (95% CI)	Bootstrap-corrected performance (95% CI)
C-statistic (AuROC)	0.929 (0.903, 0.954)	0.929 (0.905, 0.956)
O:E ratio	1.000	1.002 (0.930, 1.070)
CITL	0.000 (−0.304, 0.304)	−0.004 (−0.324, 0.323)
Calibration slope	1.000 (0.801, 1.199)	0.997 (0.776, 1.232)
Bootstrap shrinkage	-	0.997

AuROC: area under the receiver operating characteristic curve; CI: confidence interval; CITL: calibration-in-the-large; O:E ratio: observed-to-expected outcomes ratio.

### Classification performance at a predicted probability threshold of 0.471

**Figure 4:**
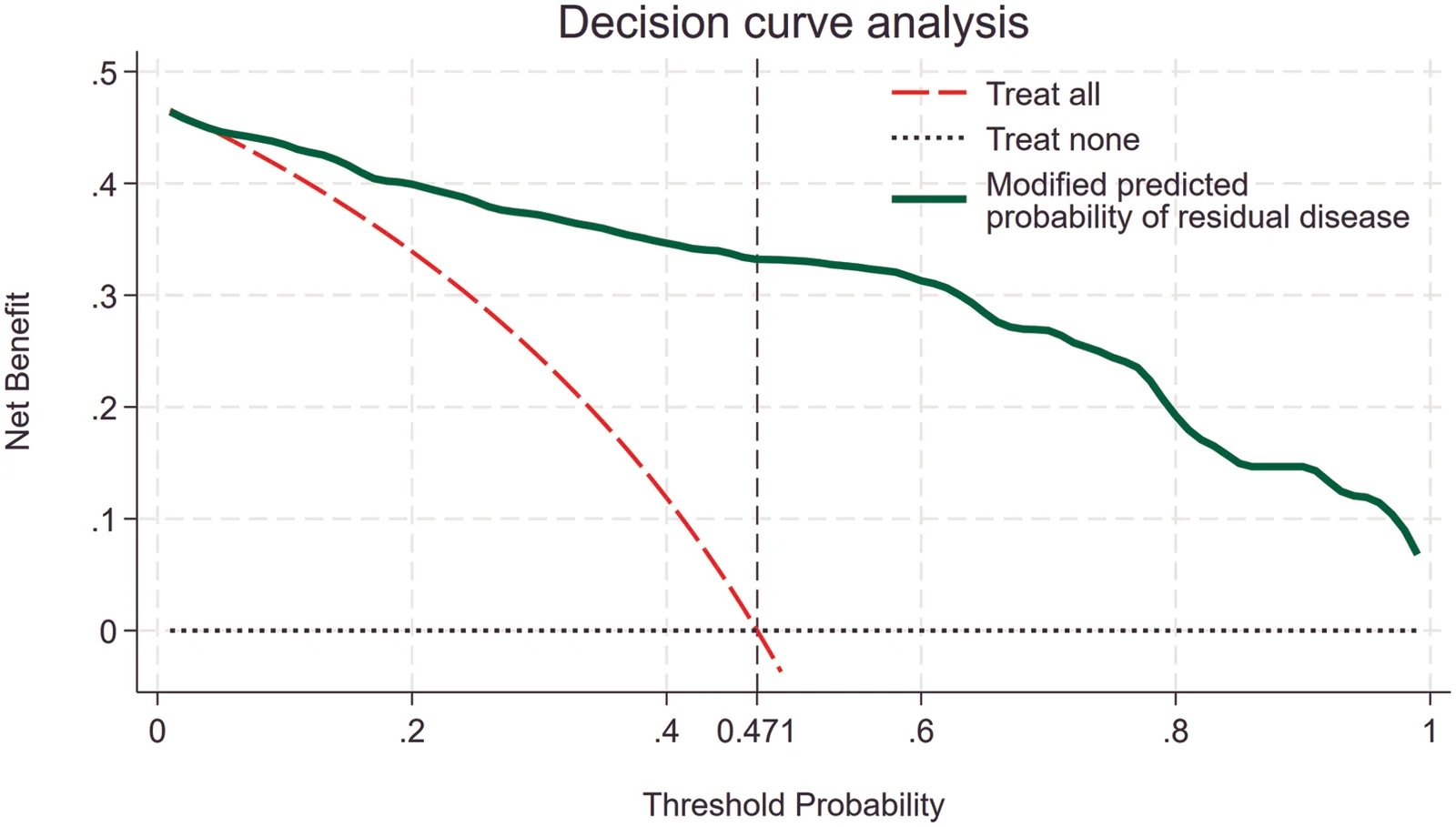
Decision curve analysis demonstrating the net clinical benefit of the prediction model for residual disease.

DCA (Fig. 4) suggested that this clinical prediction model offers greater net clinical benefit than adopting a default “treat-all” or “treat-none” approach in almost every threshold probability. For clinical use, we stratified the risk using a probability cutoff of 0.471. This threshold resulted in adequate classification of patients, with a PPV of 81.2 (95% CI: 75.1–86.4) and an NPV of 89.2 (95% CI: 83.8–93.3) for detection of RD (Table 4).

**Table 4:** Classification Performance at a Predicted Probability Threshold of 0.471

Model probability of residual disease	Residual disease (n = 180), n (% of total cohort)	Complete response (n = 202), n (% of total cohort)	PPV (%) (95% CI)	NPV (%) (95% CI)
≥ 0.471	160 (41.9)	37 (9.7)	81.2 (75.1, 86.4)	89.2 (83.8, 93.3)
< 0.471	20 (5.2)	165 (43.2)		

Percentages are cell percentages calculated using the total study population (N = 382) as the denominator. CI: confidence interval; NPV: negative predictive value; PPV: positive predictive value.

**Figure 5:**
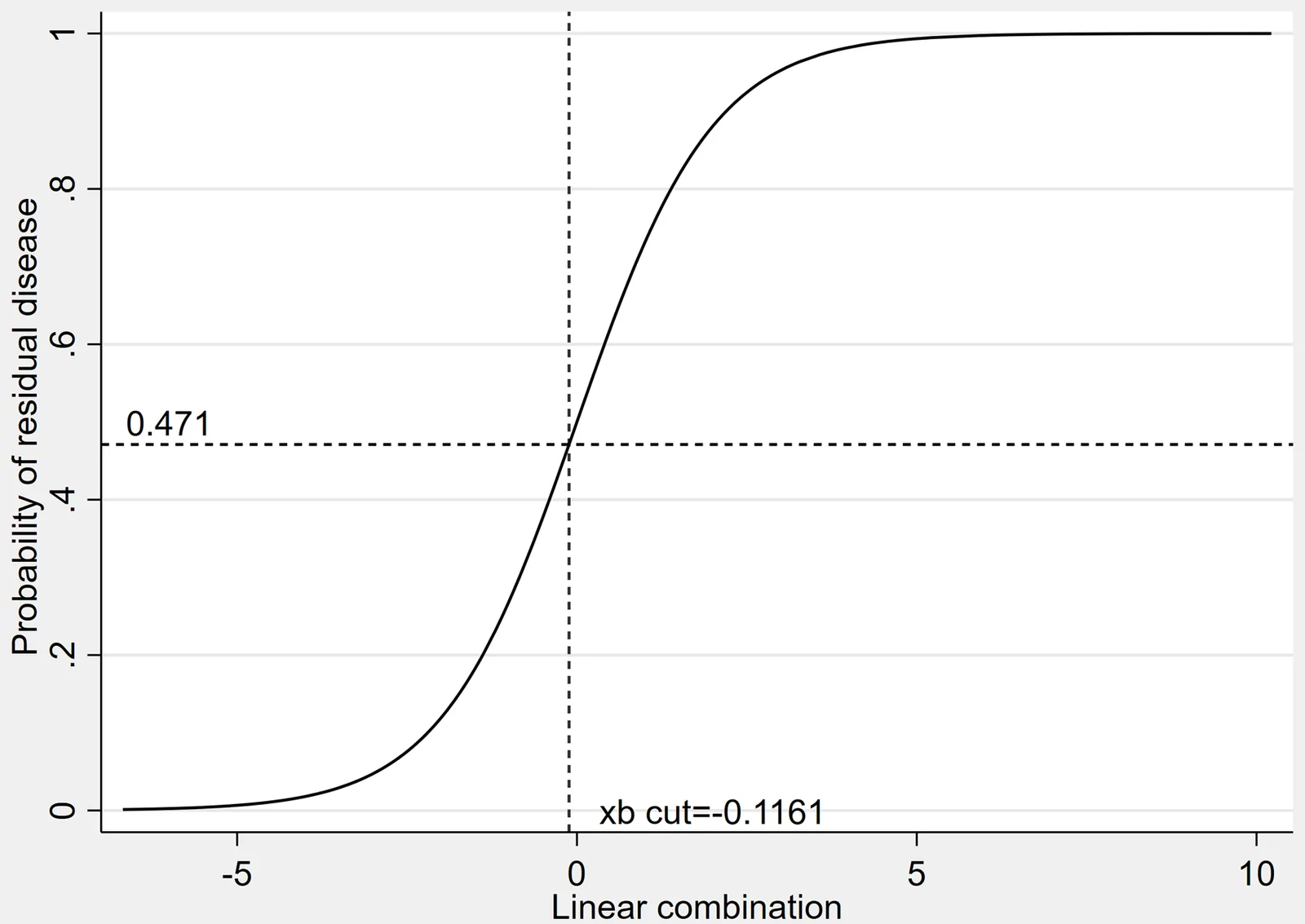
The predicted probability of residual disease (RD) plotted against the model’s linear predictor (xb), highlighting the threshold used for clinical decision-making. The linear predictor cutoff (xb cut) of −0.1161 corresponds to a predicted probability of 0.471.

Thus, patients with a linear predictor of ≥ −0.1161 (predicted probability ≥ 0.471) were categorized as being at high risk for RD (Fig. 5). A linear predictor of the model yields an estimated probability of RD. Based on the study prevalence, a prediction probability ≥ 0.471 (47.1%) was chosen as the classification threshold, which corresponded to a linear predictor cutoff of −0.1161.

## Discussion

The study utilized data from 382 patients with HNC treated with definitive CCRT, of whom 47.1% had RD. The high rate may be due to disease burden; the median maximum tumor diameter was 6.5 cm in patients from the RD group, with a significantly higher rate of T4 cases reported in that group. We developed a clinical prediction model based on readily available pretreatment variables, including tumor site, tumor burden, and time to initiation of radiotherapy. Lower BMI and hemoglobin levels were associated with RD in univariable analysis and were retained in the final model. The model demonstrated excellent discrimination (AuROC, 0.929) and good PPV and NPV to enable clinically relevant risk stratification.

Among the predictors, the tumor-intrinsic factors (tumor site and size) were most strongly associated with RD in the multivariable analysis, with hypopharyngeal origin and maximum tumor diameter being the dominant contributors. In our analysis, tumor site was a significant predictor of RD after CCRT, with hypopharyngeal cancer showing a higher risk than supraglottic tumors. These subsites are associated with poorer locoregional control and survival for hypopharyngeal and oral cavity cancers than for laryngeal and oropharyngeal subsites. These differences are probably due to the more aggressive biology of the tumor and advanced stage at presentation that are also seen in these anatomical sites. However, poor outcomes and a high risk of treatment failure in advanced hypopharyngeal cancer have been well documented in previous studies. A literature review suggests that challenging subgroups undergoing surgical resection may have a median disease-free survival of only 17.6 months [12]. Moreover, RD after CCRT is strongly predictive of overall progression-free survival (PFS). In patients with hypopharyngeal cancer, RD often results in a markedly impaired PFS with an early failure pattern [13]. With respect to regional nodal involvement, RD in our cohort was predominantly located at the primary site, whereas isolated nodal RD was uncommon. Most patients with nodal RD also had concurrent primary-site RD. This pattern is consistent with the composite locoregional definition of RD used in this study and supports modeling RD at the patient level, given the limited number of isolated nodal events.

In our analysis, the maximum tumor diameter was one of the strongest univariable predictors of RD. The strong association between a larger tumor burden and RD persists, as observed in other studies, demonstrating that an increased tumor volume is associated with adverse outcomes post-definitive CCRT [14]. Furthermore, evidence from other malignancies indicates that tumor size and volume are significant predictors of treatment response and that tumor burden is biologically important as a determinant of RD [15]. Our findings support the maximum tumor diameter as a predictor in this study and provide a biological rationale for its inclusion in the clinical prediction model of RD in locally advanced HNC based on pretreatment clinical tumor characteristics.

Temporal factors, specifically the time to initiation of radiotherapy, also contributed to the risk of RD. In our clinical prediction model, the duration between histopathological diagnosis and initiation of radiotherapy was associated with an increased risk of RD. Although population-based data suggest that the survival impact of treatment delay is site-specific, adversely affecting oral cavity, oropharyngeal, and laryngeal cancers but not necessarily hypopharyngeal cancer, our study highlights a different aspect of local control in hypopharyngeal cancer. Specifically, we found that a prolonged time to radiotherapy initiation was associated with a higher risk of RD, supporting that treatment delay directly contributes to early treatment failure [16].

In our primary analysis of risk factors for RD, we demonstrated that a prolonged time to the initiation of radiotherapy was independently associated with the risk, which implies a definitive relationship between treatment delay and early treatment failure. While site-specific survival effects of delayed treatment have been reported in population-based studies, our data elucidate that local disease control, as reflected by the presence of RD, can be adversely impacted even when no measurable survival difference is seen [17]. It is important to distinguish between two related but separate concepts. Accelerated tumor repopulation is a radiobiological phenomenon associated with prolonged overall treatment time during radiotherapy, whereas the variable examined in our study was the waiting interval before treatment initiation. Thus, the more relevant explanation for our findings is disease progression during the prolonged pretreatment interval, which may increase tumor burden before radiotherapy begins, consistent with evidence that delayed treatment initiation may adversely affect outcomes [18]. Prolonged waiting times before radiotherapy are common in developing countries and resource-limited settings because of limited radiotherapy capacity [19], and supportive procedures such as tracheostomy or feeding-tube insertion may further contribute to this delay in real-world practice. Beyond treatment-related timing factors, baseline hematologic and nutritional status also contributed to the risk of RD in our cohort. This finding is consistent with that of prior studies, which have demonstrated that baseline hemoglobin levels serve as a significant prognostic factor for OS in patients with HNSCC [20]. Pretreatment anemia has been associated with inferior survival compared with non-anemic patients [21]. Likewise, in rectal cancer, patients with pretreatment anemia who receive neoadjuvant CCRT have been shown to have decreased pathologic CR rates, with multivariate analysis confirming pretreatment anemia as an independent predictor of impaired local control [22].

Host and systemic factors, including BMI, nutritional status, hemoglobin level, and the NLR, provided additional prognostic information. The effects of BMI and malnutrition on cancer treatment outcomes have been extensively studied. Consistent with these data, a prospective radiotherapy cohort reported a significantly greater malnutrition risk in patients with stage III disease [23]. Moreover, nutritional status appraised by means of the Nutritional Risk Screening (NRS) is directly related to treatment efficiency: patients reaching a complete or partial outcome from radiotherapy usually show NRS ≤ 3, while non-responders exhibit greater rates of NRS ≥ 3. Pretreatment weight loss is clearly correlated with a higher risk of malnutrition and is an independent predictor of an inferior response to radiotherapy [23].

Importantly, NLR was retained in the model, highlighting the relevance of systemic inflammation in further malignancy progression and the development of treatment resistance. This aligns with the previously demonstrated literature that an increased pretreatment NLR was predictive of poor survival and reduced treatment response across several advanced malignancies. A recent meta-analysis found that high NLR was strongly linked to lower disease control rates during immunotherapy. This association may represent the adverse impact of systemic inflammation, in which chronically elevated neutrophil levels suppress antitumor immunity and promote resistance. Thus, pretreatment NLR is an easily accessible and clinically relevant prognostic marker before starting definitive treatment [24].

While HPV DNA is prognostic of treatment outcomes in HNC, especially oropharyngeal cancer, it is not routinely evaluated across subsites. Thus, HPV data in our study were incomplete because testing was not performed uniformly across all tumor subsites. Although oropharyngeal tumors were relatively common in our cohort (n = 177), HPV testing within this subgroup was not systematic and remained incomplete. Therefore, a sensitivity analysis restricted to the oropharyngeal subgroup and stratified by HPV status was not feasible without introducing selection bias due to incomplete and non-random HPV data. In addition, the aim of this initial model was to identify pretreatment predictors applicable across all HNC subsites, rather than biomarkers such as HPV status that are mainly informative for oropharyngeal cancer. Despite this limitation, the prediction model achieved an AuROC of 0.929, indicating high performance. The future applicability of such biomarkers remains to be determined, but the detection of circulating HPV DNA in a previous study showed promise as a stable biomarker for predicting treatment response to radical CCRT [25]. These results of HPV status in risk stratification suggest that viral biomarkers could be added to clinical prediction models in subsequent studies. In addition, recent literature has reported the performance of radiomics-based approaches for outcome prediction in HNC. Moreover, the integration of multimodal pretreatment imaging and cone-beam CT-derived features that closely resemble in-treatment anatomical changes remains a promising direction for improving predictive models [26, 27]. Although these techniques successfully characterize tumor heterogeneity and treatment monitoring, the requirement for sophisticated imaging methods and intricate feature extraction may hinder their widespread clinical application. In comparison, our prediction model is based on commonly accessible pretreatment factors, which enhances its pragmatic applicability in real-world clinical practice. While promising performance in identifying RD for nasopharyngeal carcinoma and achieving stratification of patients who may benefit from neoadjuvant chemotherapy has been reported with deep learning-based approaches using pretreatment MRI, their clinical application might be hindered due to model specificity to subsites of the disease, an analysis that requires imaging expertise, and the complexity of overall models [9]. In contrast, our clinical prediction model offers a widely generalizable and interpretable approach to predict the probability of RD across different HNSCC subsites. Our model is highly practical for everyday use because it uses only routine pretreatment clinical evaluation.

The apparent AuROC of 0.929 was relatively high for a clinical prediction model, and we therefore considered the possibility of optimism and overfitting. On internal validation using bootstrap resampling, the optimism-corrected C-statistic remained 0.929 (95% CI: 0.905–0.956), with a bootstrap shrinkage factor of 0.997 and a calibration slope of 0.997, indicating minimal optimism and no meaningful overfitting. The high discrimination may partly reflect the selected case-mix of our cohort, which consisted exclusively of patients with unresectable, locally advanced HNSCC treated with definitive CCRT. Within this high-risk population, substantial variation in pretreatment tumor burden remained, allowing strong predictors such as maximum tumor diameter and tumor subsite to discriminate well between residual and complete responders. This level of discrimination, although relatively high for a clinical model, is not unprecedented in HNC response-prediction studies. For example, a pretreatment MRI-based deep learning model for locally advanced nasopharyngeal carcinoma reported an average AUC of 0.928 for predicting residual tumor after CCRT, while prior work in locally advanced HNC showed that pretreatment tumor volume strongly discriminated early responders from non-responders [9, 14]. Nevertheless, external validation in independent cohorts remains necessary before routine clinical application.

Extranodal extension (ENE), previously referred to as extracapsular extension (ECE), is an important adverse nodal feature and has been incorporated into the AJCC eighth edition nodal staging system for several non-HPV-associated head and neck subsites. Although pathological assessment remains the reference standard, pretreatment CT and MRI may provide supportive evidence of ENE. A systematic review and diagnostic meta-analysis by Park et al reported pooled sensitivity and specificity of 73% and 83% for CT, and 60% and 96% for MRI, respectively [28]. These findings suggest that radiologic ENE may be clinically useful, although diagnostic performance remains imperfect. In this study, only pretreatment predictors were considered, and pathological ENE was not available because the cohort consisted of unresectable patients treated non-surgically. Furthermore, pretreatment ENE assessment was not standardized across this retrospective cohort because patients were referred from multiple centers and clinicians, which may have introduced heterogeneity and risk of misclassification. ENE was therefore not incorporated as a predictor in the current model. Standardized or pathologically validated ENE assessment should be evaluated in future prediction models.

### Limitation

This study has several limitations. First, its retrospective, single-center design may introduce selection bias and limit generalizability to other populations. Second, HPV status was not available for all patients, as testing was not performed uniformly across tumor subsites, so this prognostic factor could not be fully incorporated. Third, the outcome was early treatment response assessed at 8–12 weeks rather than long-term locoregional control or survival. Future multicenter, prospective studies are warranted to confirm the clinical utility of the proposed model. In addition, because tumor response was assessed at a fixed 8- to 12-week window after completion of CCRT, a subset of tumors with delayed response beyond this interval may have been classified as RD. Serial posttreatment assessment may improve response classification in future studies. Furthermore, RD was modeled as a composite locoregional outcome. Separate prediction models for primary-site and nodal RD were not developed because isolated nodal RD was infrequent in our cohort, and site-specific modeling would likely have resulted in unstable estimates. Future studies with larger cohorts should evaluate differential prediction of primary-site and nodal RD. The number of regional lymph nodes was not consistently and reliably documented in the retrospective medical records and imaging reports and therefore could not be analyzed robustly. Because this study aimed to develop a pretreatment prediction model using variables that were routinely and consistently available before treatment initiation, lymph node count was not included. Future prospective studies incorporating more detailed nodal parameters are warranted, as prior studies have often focused more on nodal metastasis detection than on lymph node counts [29]. Finally, time-to-event outcomes such as PFS were beyond the scope of the present analysis and should be evaluated in future studies.

### Conclusions

The clinical prediction model developed in this study predicts the probability of RD after completion of definitive CCRT in patients with advanced HNSCC. The model with routinely obtainable pretreatment clinical factors achieved excellent discriminative performance and high predictive values. Notably, the implementation of such a model may better individualize decision-making, enabling clinicians to identify high-risk patients who might benefit from alternative strategies (i.e., neoadjuvant chemotherapy, targeted therapy, or earlier surgical management). On the other hand, it could help prevent low-risk patients from undergoing unnecessarily aggressive treatments.

## Data Availability

The datasets generated and analyzed during the current study are not publicly available due to patient privacy constraints but are available from the corresponding author upon reasonable request.

## Abbreviations

AJCC: American Joint Committee on Cancer
AUC: area under the curve
AuROC: area under the receiver operating characteristic curve
BMI: body mass index
CCRT: concurrent chemoradiotherapy
CI: confidence interval
CITL: calibration-in-the-large
CR: complete response
CT: computed tomography
CTV: clinical target volume
DCA: decision curve analysis
ECE: extracapsular extension
ENE: extranodal extension
GTV: gross tumor volume
HNC: head and neck cancer
HNSCC: head and neck squamous cell carcinoma
HPV: human papillomavirus
IMRT: intensity-modulated radiation therapy
IQR: interquartile range
IRB: Institutional Review Board
mOR: multivariable odds ratio
MRI: magnetic resonance imaging
NB: net benefit
NLR: neutrophil-to-lymphocyte ratio
NPV: negative predictive value
NRS: Nutritional Risk Screening
O:E ratio: observed-to-expected ratio
OR: odds ratio
OS: overall survival
PD: progressive disease
PFS: progression-free survival
PLR: platelet-to-lymphocyte ratio
PPV: positive predictive value
PR: partial response
RD: residual disease
RECIST: Response Evaluation Criteria in Solid Tumors
SCC: squamous cell carcinoma
SD: stable disease
SD: standard deviation
TCTR: Thai Clinical Trials Registry
uOR: univariable odds ratio
